# Global Genetics Research in Prostate Cancer: A Text Mining and Computational Network Theory Approach

**DOI:** 10.3389/fgene.2019.00070

**Published:** 2019-02-14

**Authors:** Md. Facihul Azam, Aliyu Musa, Matthias Dehmer, Olli P. Yli-Harja, Frank Emmert-Streib

**Affiliations:** ^1^Predictive Society and Data Analysis Lab, Faculty of Information Technology and Communication Sciences, Tampere University, Tampere, Finland; ^2^Institute of Biosciences and Medical Technology, Tampere, Finland; ^3^Faculty for Management, Institute for Intelligent Production, University of Applied Sciences Upper Austria, Steyr, Austria; ^4^Department of Mechatronics and Biomedical Computer Science, UMIT, Hall in Tyrol, Austria; ^5^College of Computer and Control Engineering, Nankai University, Tianjin, China; ^6^Computational Systems Biology, Faculty of Biomedical Engineering, Tampere University, Tampere, Finland; ^7^Institute for Systems Biology, Seattle, WA, United States

**Keywords:** prostate cancer, text mining, natural language processing, network science, genetics, biomedical text mining, computational network theory, meta-analysis

## Abstract

Prostate cancer is the most common cancer type in men in Finland and second worldwide. In this paper, we analyze almost 150, 000 published papers about prostate cancer, authored by ten thousands of scientists worldwide, with an integrated text mining and computational network theory approach. We demonstrate how to integrate text mining with network analysis investigating research contributions of countries and collaborations within and between countries. Furthermore, we study the time evolution of individually and collectively studied genes. Finally, we investigate a collaboration network of Finland and compare studied genes with globally studied genes in prostate cancer genetics. Overall, our results provide a global overview of prostate cancer research in genetics. In addition, we present a specific discussion for Finland. Our results shed light on trends within the last 30 years and are useful for translational researchers within the full range from genetics to public health management and health policy.

## 1. Introduction

Cancer is not a single, homogeneous disease that is not caused by a single gene but by multiple genes and for this reason forms a complex disease rather than a Mendelian disease (Botstein and Risch, 2003; Loscalzo et al., 2007; Altshuler et al., 2008). Cancer cells grow inside the body involving an abnormal growth of cells because malfunctioning of genes leads to a denial of apoptosis preventing cells to die. As a result, cells are growing continuously and are forming tumors that turn into cancer in the human body. Cancer can have many causes like diet and lifestyle, inherited gene mutations or environmental hazards, e.g., UV radiation (American Cancer Society, 2018a,b; Cancer Research UK, 2018). Prostate cancer is the most common cancer type in men in Finland and second worldwide and in the United States. According to statistical data from Ferlay et al. (2015) there are 30.7 (World), 98.2 (USA), and 96.6 (Finland) ASR (age-standardized rate per 100, 000 population) new prostate cancer cases and 7.8 (World), 9.8 (USA), and 12.0 (Finland) ASR deaths from prostate cancer.

For this reason, it is not surprising that compared to many other types of human cancer, there is an overwhelming number of research articles on prostate cancer in the scientific literature available (Friedman et al., 2015; Dataset, 2018). For instance, PubMed (https://www.ncbi.nlm.nih.gov/pubmed/), a repository developed and maintained by the National Center for Biotechnology Information (NCBI), provides free access to research articles on prostate cancer from 1949 to 2018 and a keyword search reveals that there are almost 150,000 articles available. This huge amount of research articles provides a very important source for knowledge discovery for all kinds of interrogations of prostate cancer, e.g., cancer treatment, detection and the prevention of cancer. In this context, text mining plays a marvelous role in knowledge mining, biomedical entity recognition, gene-cancer relation identification and drug discovery.

Specifically, data mining can be used to discover knowledge from big and small data sets with the application of methods from artificial intelligence and machine learning (Vapnik, 1995; Izenman, 2008; Haste et al., 2009; Ye et al., 2016; Jensen et al., 2017; Emmert-Streib and Dehmer, 2019). One kind of data mining method for extracting information from text sources is called text mining or natural language processing (Manning et al., 1999; Collobert et al., 2011; Jurafsky and Martin, 2014). Many studies have applied text mining in the field of biomedicine to extract valuable information, e.g., about electronic health records, chemical exposure to the human body or genotype-phenotype relations (Cohen and Hersh, 2005; Spasic et al., 2005; Cohen and Hunter, 2008; Korhonen et al., 2012; Gonzalez et al., 2015; Singhal et al., 2016; Jensen et al., 2017).

In recent years, text mining has become a very popular method in cancer research. For instance, text mining has been used to create databases for multiple cancer types containing information about, e.g., associations with miRNAs (Xie et al., 2013), methylated genes (Ongenaert et al., 2007), or disease-gene associations (Pletscher-Frankild et al., 2015). Furthermore, there are studies focusing on individual cancers only. For instance, a study by Jurca et al. (2016) explored the network of genes and the countries of interest from a large number of PubMed articles for breast cancer providing an overview. In Wang et al. (2009), a database specifically for lung cancer has been created containing associations between genes and miRNAs along with experimental evidences involved in the progression of different stages of lung carcinogenesis.

In our study, we analyze prostate cancer related publications from PubMed. We analyze almost 150,000 publications with an integrated text mining and computational network theory approach (Zweigenbaum et al., 2007; Zhu et al., 2013; Dehmer and Emmert-Streib, 2015) investigating trends in prostate cancer research worldwide, including a particular discussion about prostate cancer research in Finland. Our study demonstrates how to integrate text mining with network analysis investigating research contributions of countries and collaborations within and between countries. Furthermore, we study the time evolution of individually and collectively studied genes over the last 30 years and identify highly studied genes. Finally, we discuss prostate cancer research in Finland by investigating a collaboration network of Finland and by comparing the locally studied genes with globally studied genes in prostate cancer genetics.

Overall, our results provide a global overview of prostate cancer research in genetics and a specific discussion for Finland. Our results shed light on trends within the last 30 years and are useful for translational researchers within the full application range from genetics to public health management and health policy.

## 2. Methods

### 2.1. Text Data

**Figure 1:**
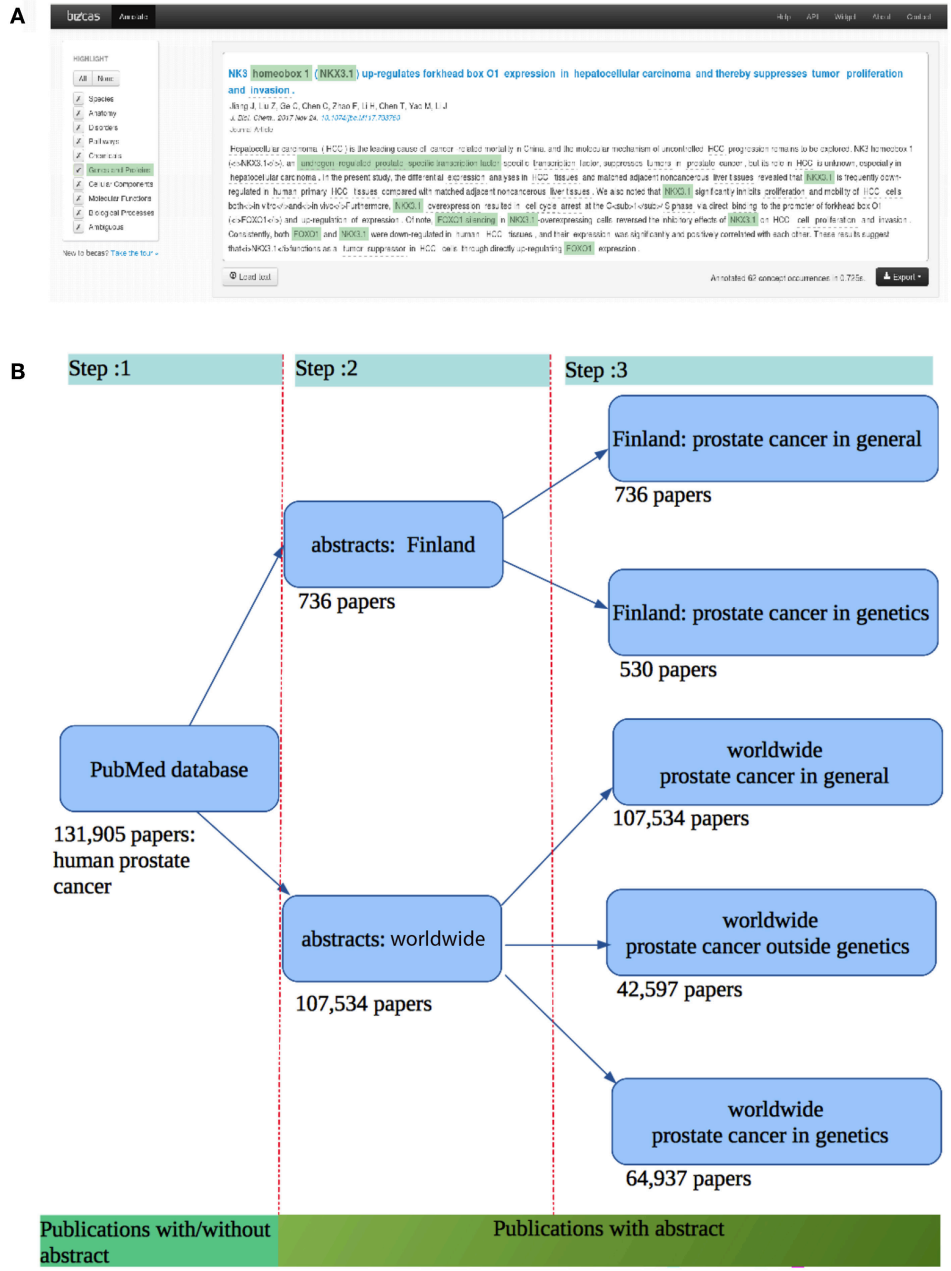
**(A)** Entity recognition step using the becas web API giving an annotation of PubMed abstracts (Nunes et al., 2013). **(B)** Data collection steps and the corresponding publications found.

For our analysis we used the becas API (Nunes et al., 2013) for information retrieval (IR) and named entity recognition (NER). In Figure 1A, we show the named entity recognition step. In this figure, the light green annotated texts passages are the Genes and Proteins identified by becas web API. We collected the PubMed ID (PMID) using PubMed API and the becas API for python client was used for retrieving all papers from PubMed. From the becas API we obtained the lexml formatted files containing biomedical annotation. The benefit of using becas API is that it annotates the genes and proteins with UniProt accession numbers, which is an unique identifier for genes and proteins. From these UniProt accession numbers, we collected all the genes and the genes that code proteins mentioned in the abstract. For this step, we use the UniProt python API to collect all the genes.

In Figure 1B, we show the corresponding steps for all data collections. First, we retrieved all the publications related to prostate cancer from PubMed. For these publications, we obtained the corresponding abstracts of the articles. Hence, our first step consists in collecting all abstracts related to prostate cancer. To retrieve these publications, we used PubMed API to the Med-line database. From this, we found 131, 905 publications related to the human prostate cancer until February 2018. After filtering out those papers which do not have an abstract, we obtained 107, 534 publications with an abstract. At step 2, we collected 107, 534 abstracts related to human prostate cancer. For this we wrote a python script to identify 736 abstracts from the 107, 534 abstracts which contain the authors' affiliation with Finland. At this step, we ended up with two different data sets: one for a Finland-centric analysis (abstract: Finland) and one for a worldwide analysis (abstracts: worldwide). At the end of step 3, we have five different data sets. The data sets for Finland (authors from Finland) is subdivided into two categories: the abstracts for prostate cancer in general (736 papers) and the abstracts for prostate cancer in genetics (530 papers). The dataset for the whole world (authors from the whole world) is subdivided into three categories: worldwide prostate cancer in general (107, 534 papers), worldwide prostate cancer in genetics (42, 597 papers), and worldwide prostate cancer outside genetics (64, 937 papers).

**Figure 2:**
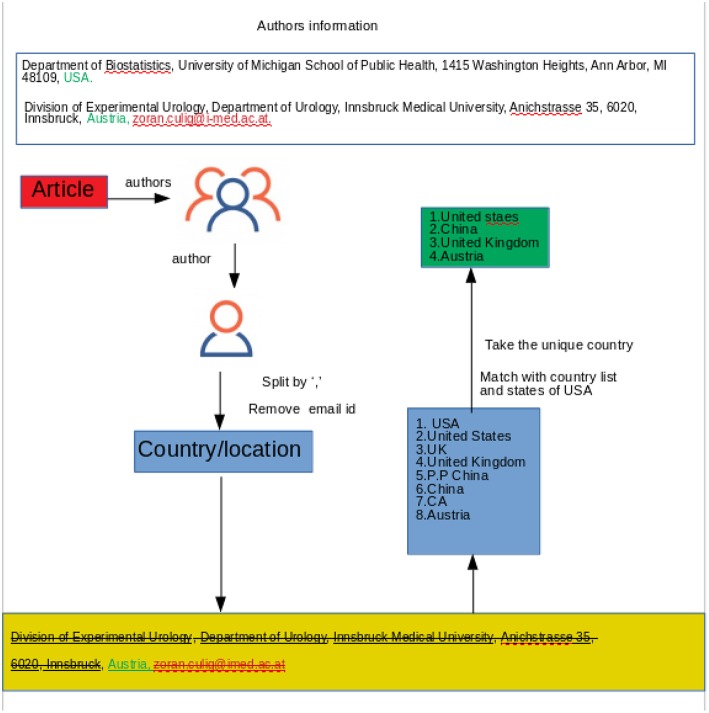
Shown is the process how to identify the country associated with an author.

For the country identification, we apply text mining instead of using Google API. Google API is a set of application programming interfaces (APIs) which allows the users to use Google services and their integration to other services. In different programming languages the users can use Google API within the code to use Google services. GeoCoding is one kind of Google API that takes the location name as an input and returns the longitude and latitude as an output. In our analysis, we first used the GeoCoding python client which takes a text (the name of the institution) as an input and returns the location information as an output (longitude, latitude, formatted address). From this formatted address, the user can identify the country. However, by using Google API we encountered several problems. Specifically, a lot of countries could not be identified due to the position of the city and the country in the paper. We noticed also that in some papers instead of the country name the author mentioned only a state or a province (for example NY instead of USA). Another reason for not using Google API is that it allows only 2, 500 queries per day, therefore information retrieval would have taken too long for the whole data set. For all these reasons, we found text mining to provide faster and more accurate results. We sub-grouped the affiliation('s) of the author('s) and we used the last part of the subgroup. Then we matched the word with a list of countries of the world and also all the states in the United States, see Figure 2. By looking at the output file from our queries, we noticed that in some abstracts, the country identification is presented as “NA” which led us to look at those specific papers for more details. After investigating the papers we found that our text mining pipeline could not identify some country names based on some locations (e.g., USA could not be identified based on the John Hopkins University). We removed those abstracts (698 abstracts), which we could not identify, the location from the authors' affiliations, from our dataset.

In order to find interesting genes studied over the prostate cancer research timeline we extracted the year of the publication and the abstract ID from the two different groups of data sets, as mentioned before.

We used Python to retrieve data and for named entity recognition from the publications. For the data analysis, we used R and the R packages igraph, NetBioV, ggplot2 are used for the data visualization. We also used Google sheet for the chord diagrams and the stack bar plots.

### 2.2. Collaboration Data

To find the collaborations between countries, we created a square matrix of the unique country names mentioned in the publications. The elements of the matrix correspond to the number of co-occurrences of pairs of countries on the publications. Due to its construction this matrix is symmetric. Summation of the rows (or columns) gives the total number of publications per country. From this, we selected, e.g., the top 10 countries which have the largest number of publications.

In order to use Google sheet for plotting the chord diagrams, we transformed the squared matrix into a three column (namely source, targets, and values) format where the source and the target represent the top ten countries and the value represents the number of co-occurrence between source and target.

### 2.3. Network Analysis

For investigating the co-occurrence of genes in publications we constructed a gene-gene literature network. In order to do this, we constructed an adjacency matrix (gene-gene co-occurrence matrix), *A*, whereas *A*_*i, j*_ ∈ ℕ gives the number of publications jointly mentioning gene *i* and gene *j*. Then we applied a threshold Θ to the elements of *A* constructing a new adjacency matrix *B* by

(1) $$B_{i,j}=\{\begin{array}{ll} 1, & \text{if }A_{i,j}>\Theta \\ 0, & \text{otherwise} \end{array}$$

We set the threshold to Θ = 10, which means that we selected only pairs of genes which were jointly mentioned in more than 10 abstracts. The reason for using this threshold is based on the estimated frequency distribution of studied genes from the literature. From this distribution, we found that

$$\begin{array}{l} Pr\left(\text{gene A and gene B are jointly studied in > 10 publications}\right)<0.0005, \end{array}$$

which is very conservative.

For the visualization of the networks, we used the R packages igraph and NetBioV (Tripathi et al., 2014).

### 2.4. Enrichment Analysis

A gene set enrichment analysis is a very popular method in computational biology and biostatistics (Emmert-Streib, 2007; Huang da et al., 2008; Tipney and Hunter, 2010). In our enrichment analysis, we investigate the portion of genes which are studied in the whole world to see whether they are enriched or not, in the three most prolific cities of Finland, see Table 1. In order to estimate the enrichment for a list of genes we need to assign two attributes to each gene. The first attribute, we call “HS” (highly studied), is having the two levels, “Yes” and “No.” The second attribute, we call “City,” is having the two levels, “In” and “Out.”

**Table 1:** Number of published articles attributed to cities of Finland.

**City**	**Publications**
Helsinki	180
Tampere	165
Turku	116
Oulu	64
Kuopio	58
Espoo	12
Jyväskylä	1

*The publications correspond to genetics research in prostate cancer and have been published between 1987 and 2018*.

Specifically, our procedure works the following way. First, we are rank ordering all genes that have been studied worldwide by their number of appearances in the publications. Second, we group this list of genes into two subcategories by introducing a threshold γ. If the number of appearances in publications for a gene is above this threshold, we place this gene in category “HS-Yes,” otherwise in category “HS-No.” That means we are distinguishing between genes that have been highly studied or not. Third, we give each gene a second attribute, “City.” If a gene has been studied in a specific “City” we give the gene the label “City-In,” otherwise “City-Out.” That means we are distinguishing between genes that have been studied in a specific city or not. As a result, each gene has now two attributes on which we base our enrichment analysis.

In Table 2 we give a formal overview of this resulting in a contingency table. Each element in the table corresponds to a count value obtained in the way described above. Here

(2) $$\begin{array}{r} n_{+1}=x+n_{21} \end{array}$$

gives the total number of genes studied in a particular city and

(3) $$\begin{array}{r} n_{+2}=n_{12}+n_{22} \end{array}$$

gives the number of genes not studied there. Similarly,

(4) $$\begin{array}{r} n_{1+}=x+n_{12} \end{array}$$

gives the total number of highly studied genes and

(5) $$\begin{array}{r} n_{2+}=n_{21}+n_{22} \end{array}$$

gives the total number of genes not highly studied. Furthermore, *n* = *n*_1+_ + *n*_2+_ = *n*_+1_ + *n*_+2_ gives the total number of genes studied worldwide.

**Table 2:**
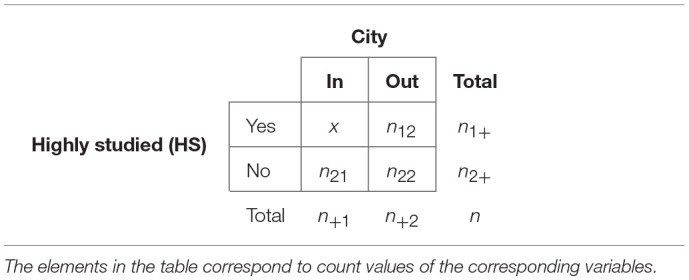
Contingency table for the enrichment analysis.

*The elements in the table correspond to count values of the corresponding variables*.

The Null Hypothesis we are studying for each city Y can be formulated by the following statement:

H_0_: The probability for a gene to be declared highly studied (“HS-yes”) and in city Y “City-In” is the same as the probability for a gene to be declared highly studied (“HS-yes”) and not in city Y “City-Out”?

The exact sampling distribution for this null hypothesis H_0_ is given by Rivals et al. (2006)

(6) $$P(x)=\frac{\left(\begin{array}{c} n_{+1}\\ x \end{array}\right)\left(\begin{array}{c} n-n_{+1}\\ n_{1+}-x \end{array}\right)}{\left(\begin{array}{c} n\\ n_{1+} \end{array}\right)}$$

From the sampling distribution, we estimate the *p*-value by

(7) $$\begin{array}{r} \text{p-value}=P\left(k>x\right)=\underset{k\in \left\{x+1,\ldots ,n_{1+}\right\}}{\sum }P\left(k\right) \end{array}$$

For assessing the statistical significance we use a significance level of α = 0.05.

## 3. Results

### 3.1. General Aspects

**Figure 3:**
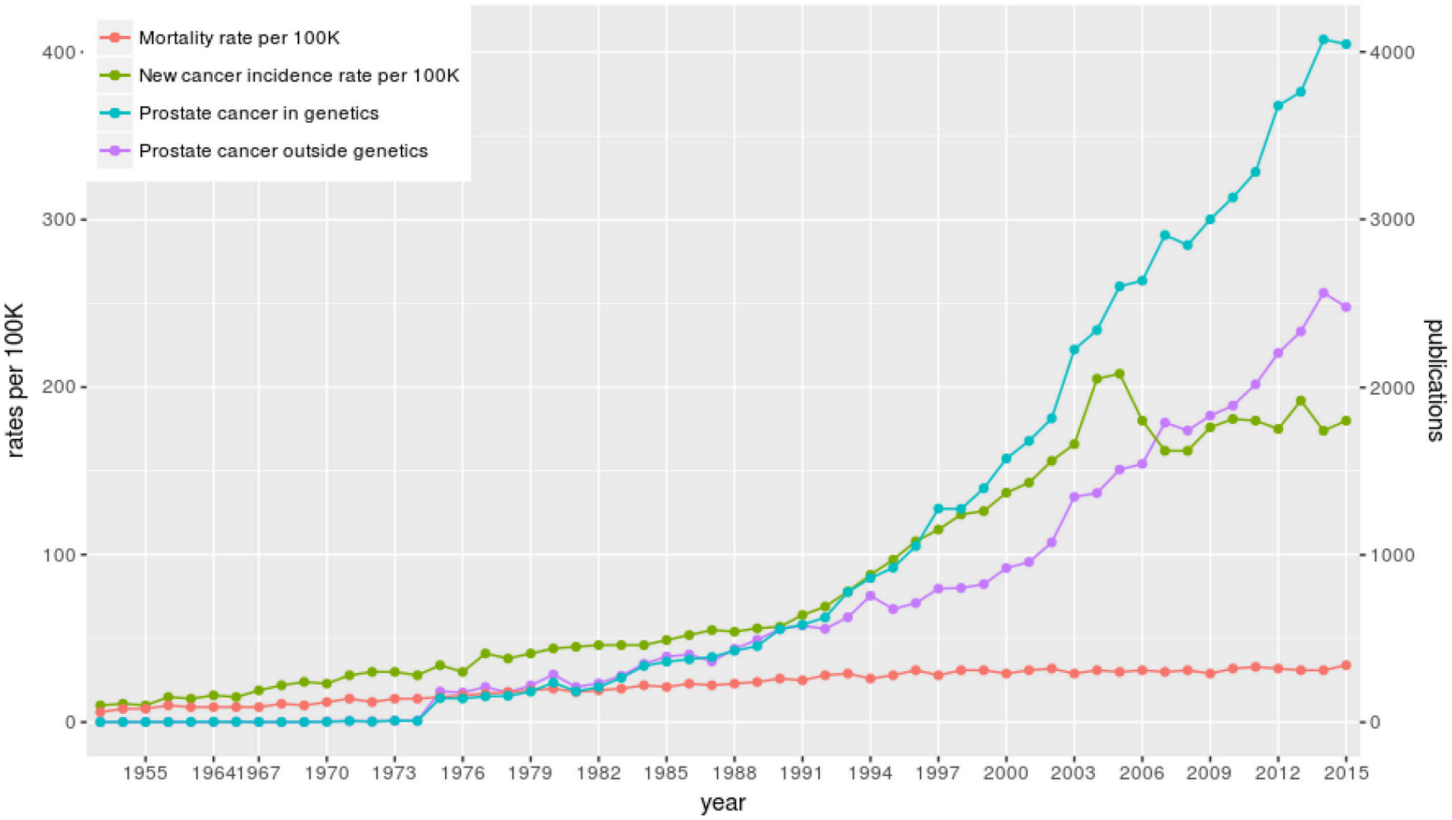
The number of published articles about prostate cancer in the world and prostate cancer statistics in Finland. The blue curve shows the number of published articles about prostate cancer in genetics and the violet curve shows the number of published articles about prostate cancer outside genetics. In red the mortality and in green the incidence rate per 100, 000 is shown for Finland.

We start our analysis by providing an overview of prostate cancer research. In Figure 3, we show the time line of the research in prostate cancer over the last 67 years (from 1949 to 2017). In this figure, we show information about the number of published articles. Specifically, we show (I) the number of published articles for prostate cancer research in genetics (blue line) and (II) the number of published articles for prostate cancer research outside genetics (violet line). The right y-axis represents the corresponding values for these two curves. This means the number of articles of type (I) and type (II) together give the total number of published articles on general prostate cancer research in the world. Here we categorize an article as “genetics research” if the abstract mentions gene names. Articles “outside” genetics research are, e.g, survival studies (Johansson et al., 1997) or public health studies about quality of life (Litwin et al., 1998).

From Figure 3, we see that the first paper about genetics research on prostate cancer appeared in 1953 (see also Table 3). The total number of published articles from all these years for all prostate cancer related studies is 131, 905 and for all prostate cancer research in genetics is 64, 937. That means there is a very large number of articles that has been published in both categories over the years and the number of articles is still increasing. From Figure 3 one can see a steady growth year-by-year and the dip for the years 2016, 2017, and 2018 is only due to the delay in the listing of the published articles in PubMed. Overall, there is a wealth of information that can be extracted from these papers and in the following, we will present results from this analysis.

**Table 3:** The table provides information about the year the first publication about a gene has been found and what country/countries contributed to this publication (see Figure 7).

**Gene**	**Country**	**Year**
KLK3	Italy	1953
KLK2	Italy	1953
ACAD9	Brazil	1965
AR	Brazil	1965
MAPK1	Brazil	1965
MAPK10	Brazil	1965
MAPK12	Brazil	1965
MAPK13	Brazil	1965
MAPK14	Brazil	1965
MAPK3	Brazil	1965
MAPK6	Brazil	1965
MAPK7	Brazil	1965
NPEPPS	Brazil	1965
MAPK8	Brazil	1965
CDKN2A	United Kingdom	1971
MSMP	United States	1973
ACTRIB	United States	1976
CBX8	United States	1976
PCSK1	United States	1976

In order to understand the increase in prostate cancer research, as discussed above, we added to Figure 3 information about prostate cancer in Finland from the year 1953 to 2015. Specifically, we show (III) the new cancer incidence rate per 100, 000 population in Finland (green line) and (IV) the mortality rate per 100, 000 population in Finland (red line) (data are from the Finnish Cancer Society and the Finnish Cancer Registry, respectively Dataset, 2018). The left y-axis represents the corresponding values for these two curves.

As one can see, over the time line, mortality and incidence rates are increasing steadily. Unfortunately, since the 1990*s* the prostate cancer incidence rate increased severely indicating many new cases year-by-year. A similar increase is observable for other countries as well (not shown). Overall, these gained rates explain the increased interest in prostate cancer research in the last 20 years.

### 3.2. Country-Specific Research Contributions

In order to obtain an overview of prostate cancer research, we study the country-specific research contributions. For this analysis, we use information about the affiliation of authors to identify the corresponding country of the institution. This allows us to identify the countries associated with a published article. In the following, we distinguish between publications about general aspects of prostate cancer and articles about the genetics of prostate cancer.

**Figure 4:**
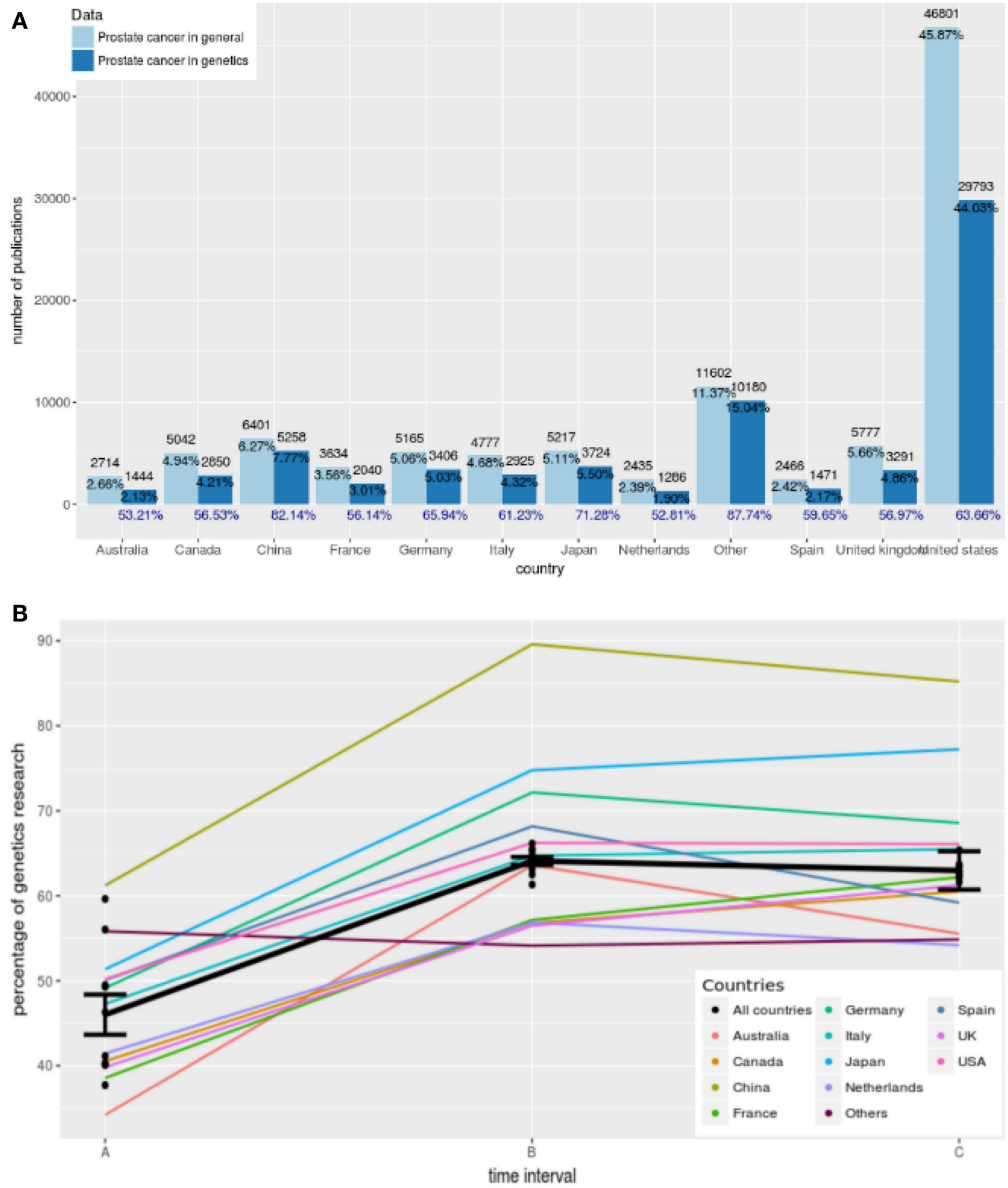
**(A)** Country-specific contributions to prostate cancer research from 12 countries. Dark blue corresponds to results for prostate cancer research in genetics and light blue corresponds to results for general prostate cancer research. The percentages in blue give the country-specific contributions to genetics research. **(B)** Trends of genetics research from 1987 to 2018 showing the percentage of genetics research. The time intervals correspond to A: 1987–1996, B: 1997–2006, and C: 2007–2018.

As a result from our text analysis, we find in total 119 different countries contributing to articles about general aspects of prostate cancer and 100 countries for articles about the genetics of prostate cancer from 1987 to 2018. Due to the fact that most countries publish only a few articles, we combine countries that contribute less than 1, 000 publications in total. We group these countries into a category we call “Others.” In Figure 4A, we show the number of articles published for the 12 most prolific countries in the world. In this figure the results for general research in prostate cancer are shown in light blue and the results for genetics research in prostate cancer are shown in dark blue. The percentages shown in black are given with reference to the total number of publications worldwide whereas the percentages in blue give the country-specific contributions to genetics research. For instance, Australia published 2, 714 articles about general research in prostate cancer which corresponds to 2.66% of all publications worldwide in this category. In contrast, Australia published 1, 444 articles about genetics research in prostate cancer which corresponds to 53.21% (= 1, 444/2, 714 × 100%) of all publications in Australia about prostate cancer research. We call this the percentage of genetics research in prostate cancer.

The country that published the largest number of research papers on prostate cancer in both cases is the United States. Specifically, authors affiliated with the United States published 46, 801 and 29, 793 articles, which correspond to 45.87 and 44.03% of all papers worldwide, respectively. Following the United States, China is ranked second with 6, 401 and 5, 258 publications, respectively. The third largest number of published articles on prostate cancer in general come from the United Kingdom whereas in the case of genetics research in prostate cancer, Japan is ranked on third position. Interestingly, the number of publications from “Others” is larger than of all other countries except the USA.

The contributions of authors from Finland to prostate cancer research in general and genetics research on prostate cancer is 736 and 530 publications, respectively, which correspond to a percentage of 0.7 and 0.8%, respectively (not shown).

The results in Figure 4A shows average values for the years 1987–2018. In order to see how genetics research evolved within this time frame we show in Figure 4B trend lines of genetics research. These trend lines are obtained in the following way. First, we estimate for every year the country-specific “percentage of genetics research.” That means, we do this separately for every country shown in Figure 4A, and for all countries together. Second, we form three time intervals A, B, and C corresponding to A: 1987–1996, B: 1997–2006, and C: 2007–2018. Third, we perform for every country two linear regressions, one for the time intervals A and B (first line) and one for the time intervals B and C (second line).

For each of the linear regressions we perform a hypothesis test for the slope of the estimated regression line, testing:

H_0_ : β_1_ = 0.

The results for this are shown in Table 4. For a significance level of α = 0.05 and a Bonferroni adjustment (we are testing 13 hypothesis), we see that for the first regression line, the slope of all of the countries but one (Others) is significant. Also, all countries together give a very strong significant value. This means most of the countries and all countries together show a significant increase in the percentage of genetics research between 1987–1996 and 1997–2006. Overall, Australia shows the largest increase because its slope is β_1_ = 29.37.

**Table 4:** Results of a linear regression analysis for Figure 4B.

**Country**	**Value of β_1_ (First line)**	***P*-value of β_1_ (First line)**	**Value of β_1_ (Second line)**	***P*-value of β_1_ (Second line)**
All countries	18.08	6.04e-07	−1.12	0.056
Australia	29.37	5.48e-05	−8.06	0.054
Canada	16.27	0.0005	3.69	0.169
China	28.38	0.0003	−4.40	0.139
France	18.59	0.018	5.038	0.242
Germany	23.00	0.001	−3.58	0.262
Italy	17.44	0.002	0.76	0.846
Japan	23.41	0.0001	2.45	0.510
Netherlands	15.41	0.02	−2.65	0.464
Others	−1.69	0.070	0.73	0.237
Spain	18.08	0.030	−8.98	0.077
UK	16.64	0.008	4.76	0.205
USA	16.03	5.9E-05	−0.14	0.884

*Shown are the values of the slope (β_1_) and the corresponding p-values*.

On the other hand, for the second regression line none of the slopes is significant that means between 1997–2006 and 2007–2018 the percentage of genetics research has not been changed significantly but remained constant. Despite not being statistically significant, Australia and Spain *reduced* their contributions to genetics largest in this time duration compared to all others.

### 3.3. Worldwide Collaborations Between Countries

In this section, we study the collaborations in prostate cancer research between countries. In order to study this, we use again information about the affiliation of authors from the articles. From these affiliations we obtain the country where an institution is located. If authors of an article are connected to more than one country, we assume that these countries are collaborating with each other. Hence, this is a direct extension of our results presented in the previous section by considering joint contributions to an article.

**Figure 5:**
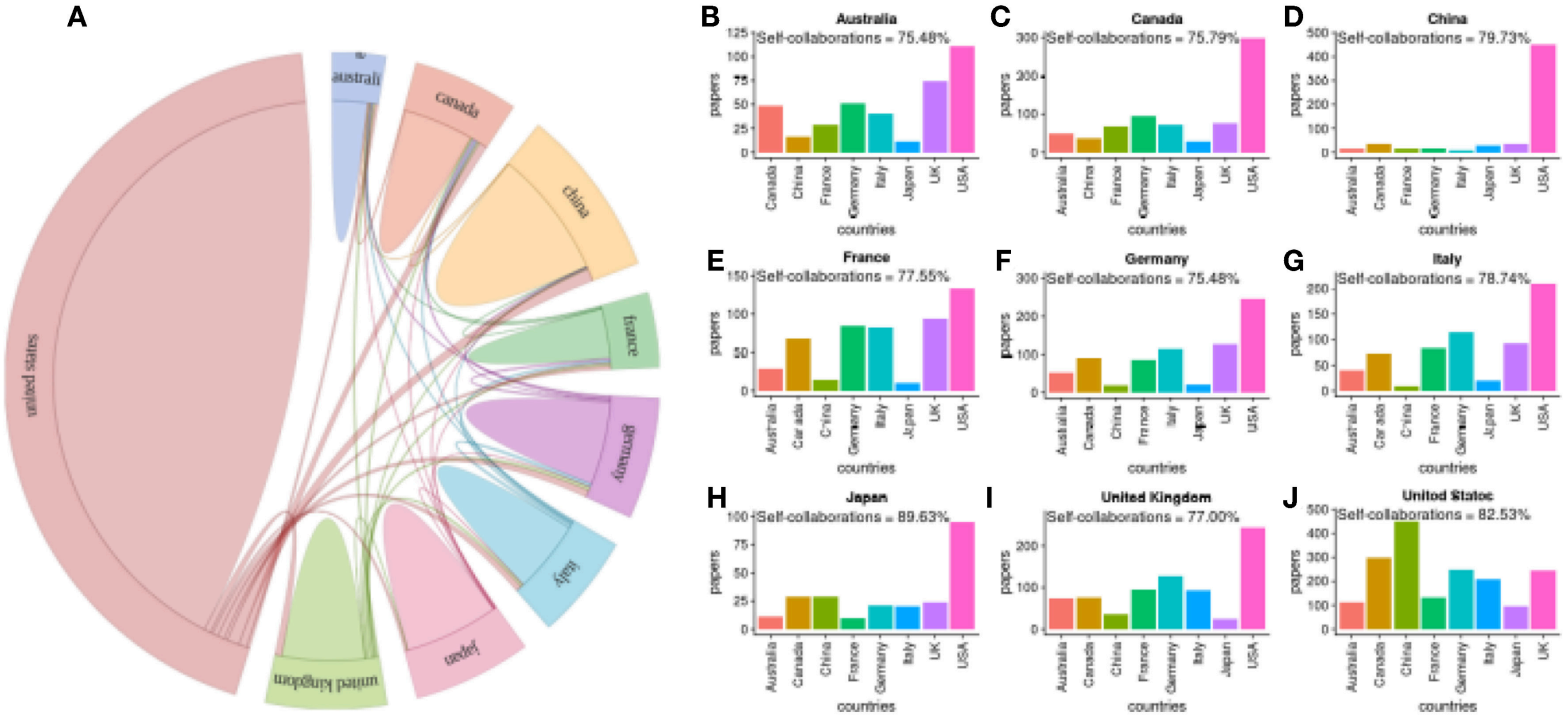
**(A)** Summary of worldwide collaborations between countries for publications about prostate cancer research in genetics. **(B–J)** Pairwise collaborations between the top nine countries. Self-collaborations give the percentage of publications with all authors from the same country.

In the following, we focus on publications about the genetics of prostate cancer. In Figure 5A, we show a global summary of worldwide collaborations between countries and in Figures 5B–J we show results of pairwise collaborations for the nine most prolific countries. We find that authors from the United States most frequently collaborate with authors from China (450 joint publications) and Canada (298 joint publications), see Figure 5J, compare to all other countries. In general, the most frequent collaboration partner of all countries is the USA. Interestingly, only China has a single major collaboration partner, namely the USA. All other countries collaborate with multiple countries.

We just want to note that by looking at the results about prostate cancer research in general (not shown), we find that also here the USA collaborates most frequently with China (523 joint publications) and Canada (465 joint publications).

### 3.4. Finland-Centric Collaborations

**Figure 6:**
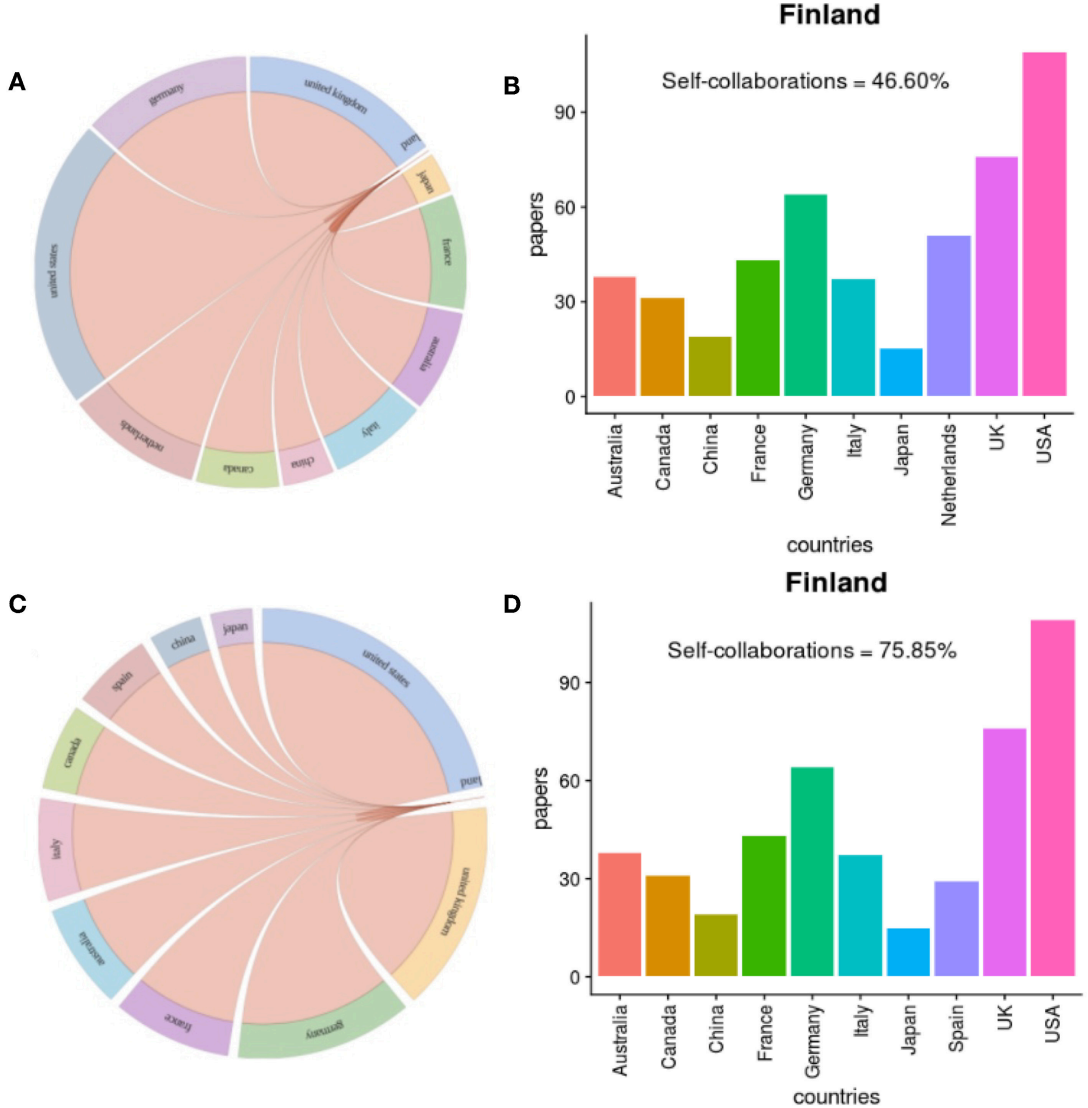
Collaborations between Finland and top 10 countries in prostate cancer research. **(A,B)** show results about prostate cancer research in general and **(C,D)** about prostate cancer research in genetics.

Next, we study the Finland-centric collaborations of authors from Finland with other countries. In total, we find that 1, 905 authors are affiliated with Finland who contribute to 736 prostate cancer articles. In Figure 6, we show the collaborations between Finland and the top 10 countries for prostate cancer research in general (Figures 6A,B), and prostate cancer research in genetics (Figures 6C,D). Figures 6B,D are the corresponding bar plots of Figures 6A,C which quantify the amount of research articles that have been published in collaboration with Finland and the top 10 countries in the world.

For prostate cancer research in general, we find a total of 736 publications that include at least one author from Finland. From these, 343 publications have only authors from Finland, which corresponds to 46.6% of all publications. For this reason, this information is not shown in Figure 6B as a bar in the plot because this exceeds the collaborations with all other countries by orders of magnitude. We see clearly that the United States is the first in rank collaborating with Finland followed by the United Kingdom and Germany. With China and Japan, Finland has less frequent collaborations.

While Sweden is not among the top 10 most prolific countries in prostate cancer research we find that Finland collaborates frequently with Sweden (47 joint publications).

For prostate cancer research in genetics, we find a total of 530 publications that include at least one author from Finland. From these, 402 publications have only authors from Finland, which corresponds to 75.85% of all publications. In Figure 6D, we see that the United States is again ranked first followed by the United Kingdom and Germany. In prostate cancer research in genetic, Spain replaces the Netherlands in collaborating with Finland, compared to Figure 6A. It is striking that Finland has only a moderate number of research collaborations with China although China has the second largest number of publications in the world. In total, Finland collaborates with 48 countries in the prostate cancer research.

Overall, prostate cancer research in Finland is very Finland-centered outnumbering the international collaborations by far. This is the case for prostate cancer research in genetics and prostate cancer research in general. Interestingly, this can be observed similarly for all other countries, see Figures 5B–J.

### 3.5. Time Evolution of Studied Gene

It is generally accepted that a single gene defect barely increases the risk or leads to cancer. Instead, it is assumed that the risk factors of cancer are due to multiple gene defects, whereas each of these genes makes a minor contribution to the increase in the risk of cancer (Pukkala et al., 2013). Over the past 30 years, many researchers were trying to find genotype-phenotype relations between genes and cancer (Gundem et al., 2015; Khanna et al., 2015; Ylipää et al., 2015; Bova et al., 2016). For this reason, in this section we identify the genes that have been studied most frequently in this period. We present results for (I) all prostate cancer studies in genetics worldwide and for (II) prostate cancer studies in genetics that have been conducted in Finland. For a publication to be counted as “conducted in Finland” it is sufficient if one author has a Finnish affiliation.

**Figure 7:**
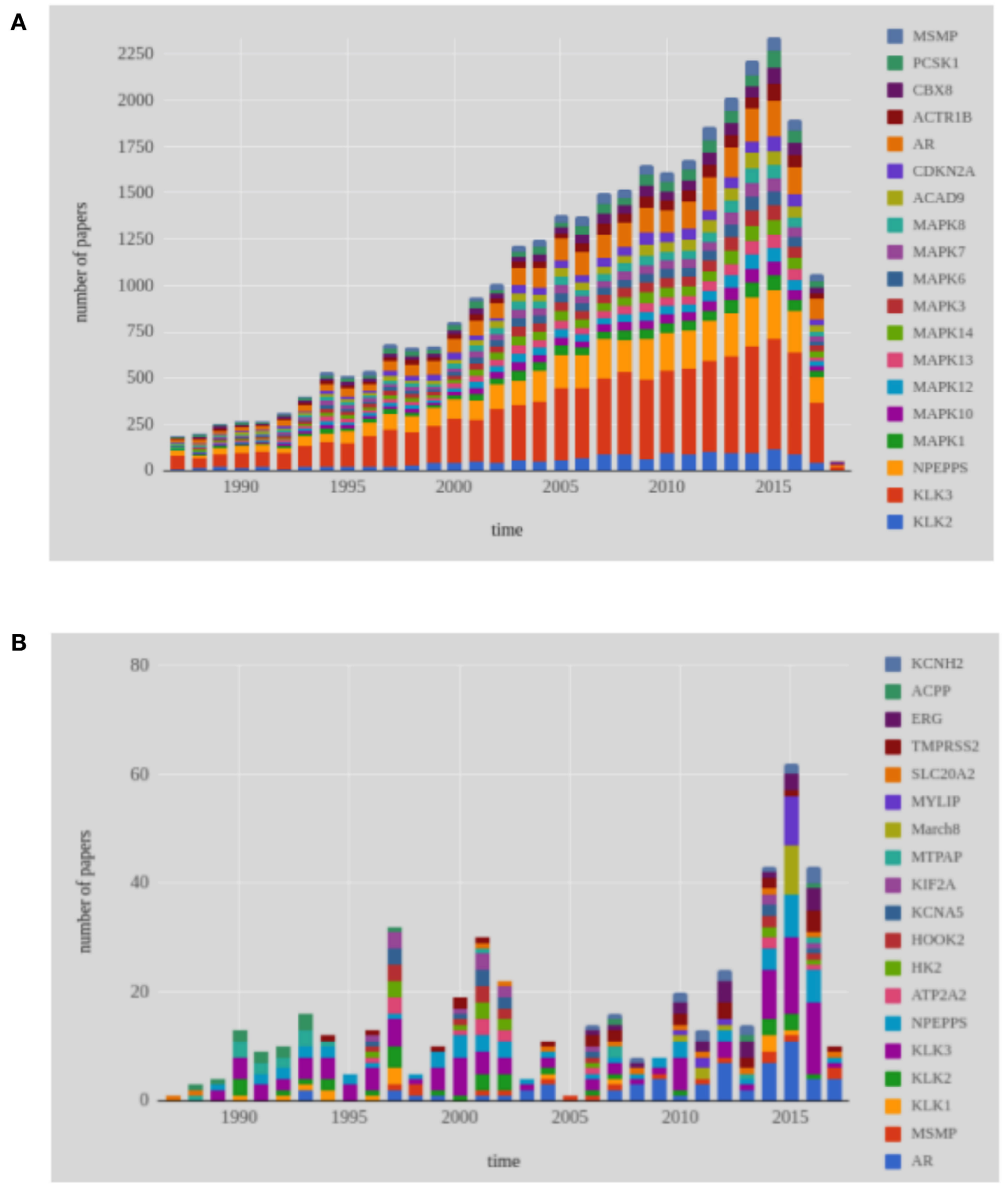
Shown are results for the 19 most frequently studied genes in prostate cancer research from 1987 to 2017. **(A)** Genes studied worldwide, **(B)** Genes studied in Finland.

In total, 7, 519 genes have been studied worldwide over the time line in genetic prostate cancer research. In Figure 7A, we show the 19 most frequently studied genes during the last 30 years. The three most frequently studied genes, KLK3, NPEPPS, and AR, have been found being continuously studied. From our data set, we have noticed that KLK3, NPEPPS, and AR have been mentioned 9,389, 4,259 and 2,895 times, respectively in the abstracts. Mitogen-Activated Protein Kinases, also known as MAP Kinases (MAPK) belongs to CMGC Kinases group. Among the 13 members of the MAPK gene family, 9 genes are among the top 19 most frequently studied genes in the world.

We performed a similar analysis for Finnish publications, see Figure 7B. In total, 1, 078 genes were studied by Finnish researchers over the last 30 years (1987–2017) in the area of prostate cancer research. The most frequently studied gene during the last 30 years is KLK3 (see Figure 7B). Following KLK3, AR and NPEPPS are the second and third most studied genes in Finland. In the year 2015, MYLIP and MARCH8 have been appeared together in nine publications. Overall, a continuous study of KLK3, NPEPPS, and AR is found from the Figure 7B.

Androgen receptor (AR) is a steroid hormone nuclear receptor family. Other members of the steroid nuclear hormone family are Estrogen Receptor (ER) and Progesterone Receptor (PR). AR is located on the X chromosome and androgen is also documented as a significant biological action on the bone, muscle and the prostate (Davey and Grossmann, 2016; Latonen et al., 2017). In many research articles, AR has been indicated as a possible cause for the progression and the development of prostate cancer (Waltering et al., 2011). The Kallikrein 3 (KLK3) gene is identified as a biomarker of prostate cancer by many researchers (Penney et al., 2011). The byproduct of KLK3 is Prostate Specific Androgen (PSA) which is used as a biomarker of prostate cancer. KLK3 is located on chromosome 19 and found to be over expressed of in men's prostate cancer cells. After the discovery of the connection between AR gene expression and prostate cancer in the year 1995 (Visakorpi et al., 1995), this attracted Finnish researchers' interest. Together with the AR gene, the NPEPPS gene has been also studied many times over the time line of our investigation.

In Table 3, we show information about the year when a gene has been studied for the first time. Specifically, for every gene in Figure 7 we identify the year it has been studied first and the country/countries that participated in this study. This complements the results shown in Figures 7A,B, because this analysis reveals explicitly the initiation of research focusing on a particular gene.

### 3.6. Prostate Cancer Research in Finland

For the rest of the paper we are focusing on prostate cancer research conducted in Finland.

#### 3.6.1. Gene-Gene Literature Network Analysis

In this section, we will analyze gene-gene networks based on the literature where authors are affiliated with Finland. Many researchers studied gene networks based on gene expression data (e.g., Werhli et al., 2006; de Matos Simoes and Emmert-Streib, 2012). However, here, we will build networks based on the literature. For this reason, we call these networks gene-gene literature networks.

**I. Top** 20 **highly studied genes**

**Figure 8:**
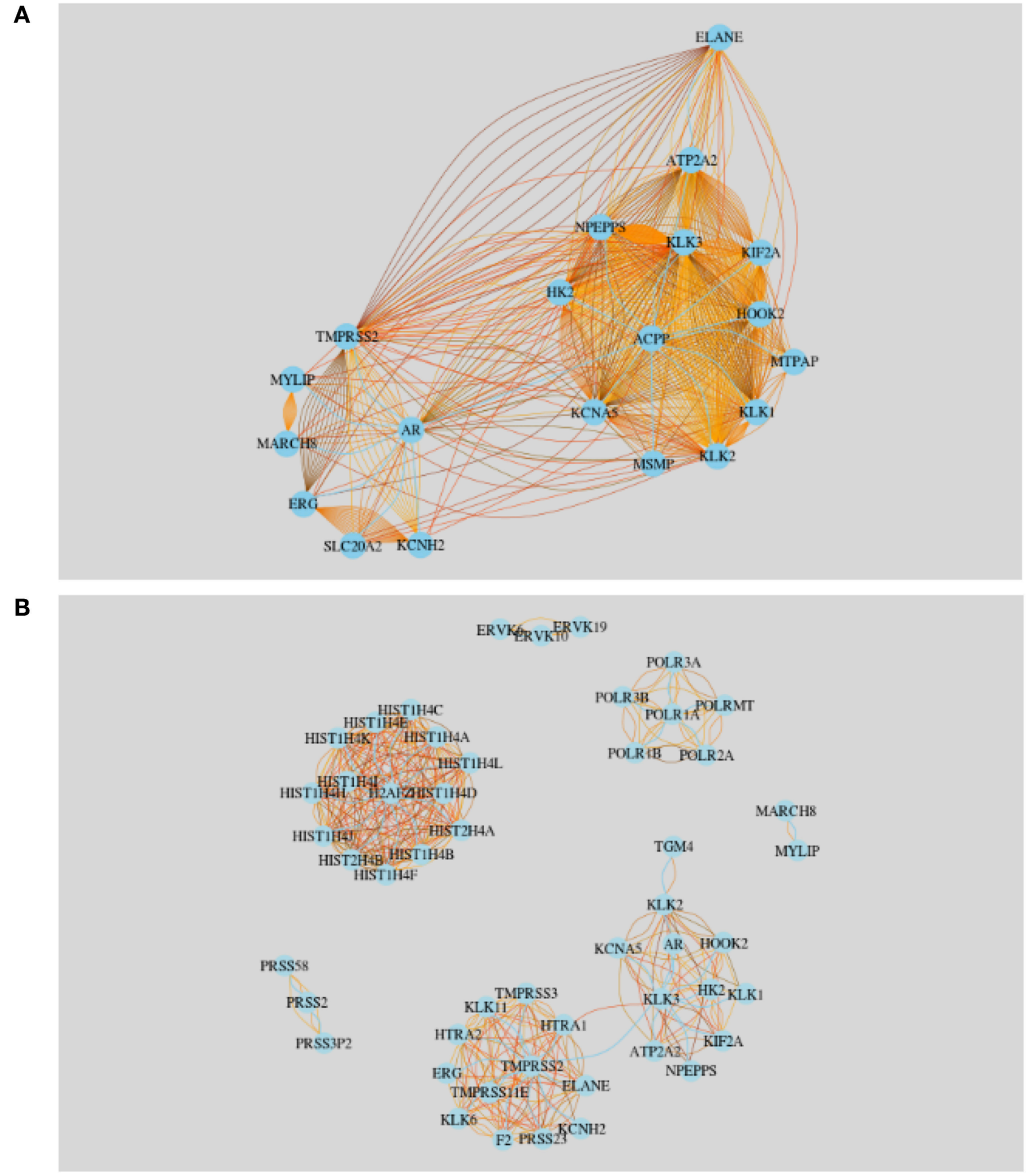
Literature based gene-gene network for Finland. **(A)** Twenty most frequently studied genes in Finland. **(B)** Community network resulting from all studied genes in Finland.

The first network we built is for the 20 most frequently studied genes in Finland. We start by creating a gene-gene co-occurrence matrix, *A*, whereas *A*_*i, j*_ ∈ ℕ gives the number publications jointly mentioning gene *i* and gene *j*. In Figure 8A we show this network. Here the nodes represent the 20 most frequently studied genes and the edges between pairs of genes represent the number of publications they appeared together. The sky blue edges represent the least amount of abstracts identified among the pairs of nodes. For example, gene AR and MYLIP have been studied together in the least number of abstracts. In another study, we have noticed that AR and NPEPPS have been studied the same amount of times over the time line which leads us to investigate the strongest connection between the top most studied gene KLK3. From Figure 8A we see that KLK3 has stronger connections with NPEPPS than with AR. From the literature data, we find that KLK3 and NPEPPS have been studied together in 52 articles in prostate cancer research in genetics among Finnish researchers.

**II. Communities in the gene-gene literature network**

Next, we extend our study beyond the 19 most frequently studied genes using all 1, 078 genes studied in Finland. That means, we construct another gene-gene literature network, however, this time for 1, 078 genes, corresponding to all genes mentioned in the publications with authors from Finland. We construct again a gene-gene co-occurrence matrix, *A*, whereas *A*_*i, j*_ ∈ ℕ gives the number publications jointly mentioning gene *i* and gene *j*. Then we apply a threshold Θ to the elements of *A* constructing a new adjacency matrix *B* by

(8) $$B_{i,j}=\{\begin{array}{ll} 1, & \text{if }A_{i,j}>\Theta \\ 0, & \text{otherwise} \end{array}$$

We set the threshold to Θ = 10, which means that we selected pairs of genes which were jointly mentioned in more than 10 publications. Overall, this corresponds to a probability of connecting two genes with more than 10 joint publications of < 0.0005 (see Methods section).

The resulting gene-gene literature network is shown in Figure 8B. In total we observe 7 communities and the largest two communities are connected by the gene KLK3, see Figure 8B. That means KLK3 acts as a bottleneck between the two largest subnetworks. See Table 5 for a summary of the genes found in the communities.

**Table 5:** Genes belonging to the communities shown in Figure 8B.

**Network**	**Gene symbols**	**Number of genes**
Network 1	AR, KLK3, NPEPPS, ATP2A2, HOOK2, KIF2A, KLK2, HK2, KCNA5, TGM4, TMPRSS2, KCNH2, HTRA1, HTRA2, ELANE, KLK6, TMPRSS11E, ERG, KLK11, PRSS23, TMPRSS3, F2	23
Network 2	HIST1H4A, HIST1H4B, HIST1H4C, HIST1H4D, HIST1H4E, HIST1H4F, HIST1H4H, HIST1H4I, HIST1H4J, HIST1H4K, HIST1H4L, HIST2H4A, HIST2H4B, H2AFZ	14
Network 3	POLR1A, POLR1B, POLR2A, POLR3A, POLR3B, POLRMT	6
Network 4	PRSS2, PRSS3P2, PRSS58	3
Network 5	ERVK19, ERVK6, ERVK10	3
Network 6	MYLIP, MARCH8	2

In Figure 8B some genes (AR,KLK3,NPEPPS) are clustered in one network. This is because researchers mentioned them pairwise in many abstracts. A possible reason why these genes are discussed many times together by many researches is that they might share the same protein domain or they might have similar co-expressions. The other large community in this figure is formed by HIST genes, which is isolated from all the other modules. The smallest community is formed by MYLIP and MARCH8. We found that these two genes were studied 14 times together in publications.

#### 3.6.2. Spacial Collaboration Network of Finland

In contrast to the previous section, now we study a network representing the collaborations between scientists in Finland. Specifically, we construct a network where nodes correspond to cities and edge between two nodes corresponds to the number of joint publications between scientists of the two cities. That means we identify for each author on a paper the city the author is located and then we eliminate from this list multiple occurring city names giving a list where each city is only mentioned once. For instance, for a paper with five authors, three from Helsinki, one from Tampere and one from Oulu, this list would include Helsinki, Tampere, and Oulu and we identify one collaboration between Helsinki and Tampere, one collaboration between Helsinki and Oulu and one collaboration between Tampere and Oulu. If there are only authors from the same city on a paper, possibly from different institutions, we count this paper as one self-collaboration. For instance, we have the Helsinki University Hospital, University of Helsinki, FIMM lab, and Biomedicum as the main institutions in the biomedical research arena that are all located in Helsinki. Overall, the resulting network contains spacial information about the location of scientists and, hence, it is a literature-based spacial collaboration network of Finland.

For this network analysis, we collected information about all laboratories and universities in Finland to which authors are affiliated. In Table 1, we show the number of publications for seven cities in Finland published for the time duration of our study (1987–2018). The top three prolific cities are Helsinki, Tampere, and Turku, whereas Helsinki has the largest number of publications on prostate cancer (see Table 1).

**Figure 9:**
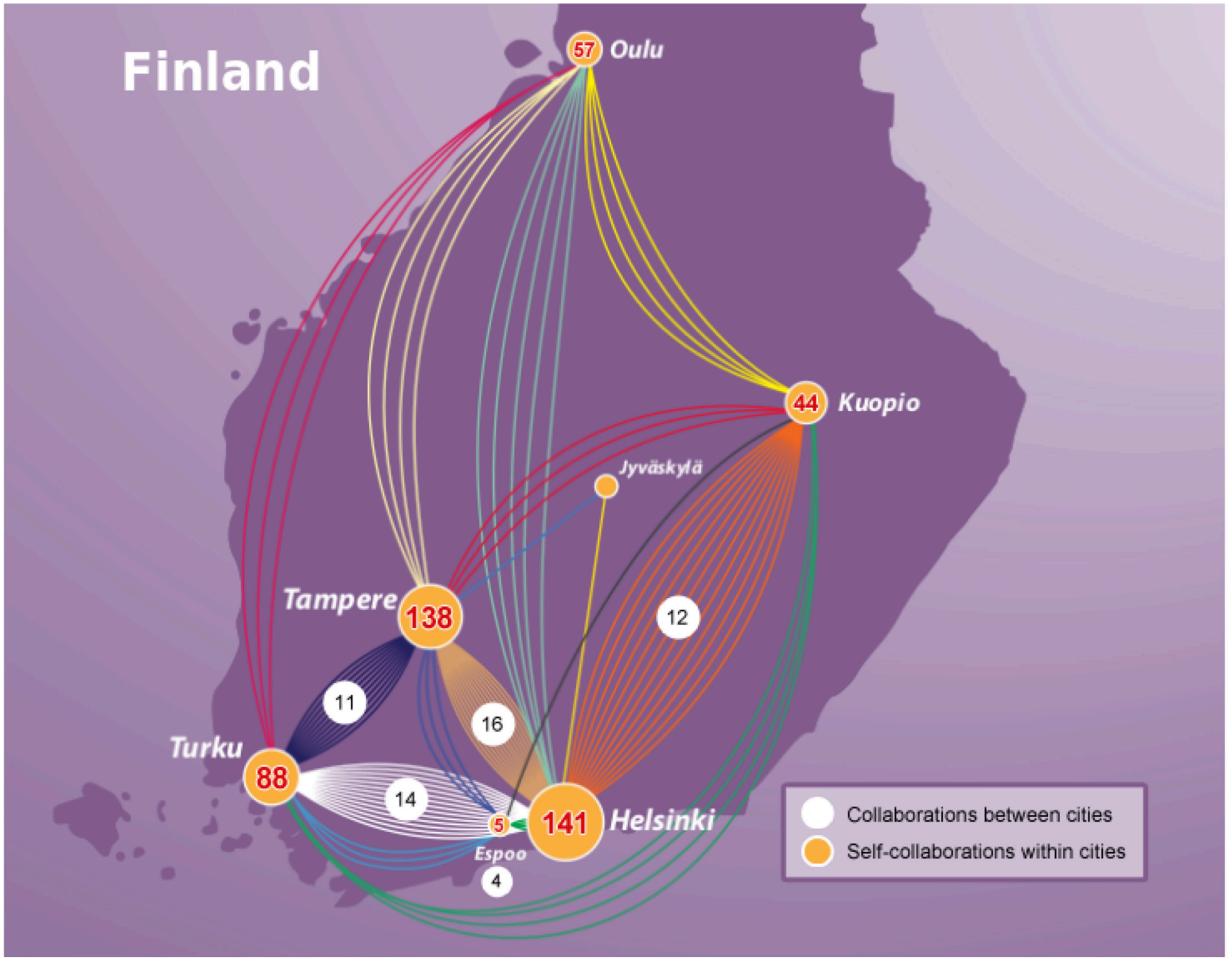
Collaboration network of Finland for genetics research in prostate cancer. Nodes correspond to cities and edges correspond to jointly published articles. The numbers provide information about the number of collaborations between cities (white background) and self-collaborations within cities (yellow background).

In Figure 9, we show the spacial collaboration network of Finland between the cities for genetics research in prostate cancer. Here the nodes of the network correspond to cities and the edges of the network correspond to the number of joint publications. One can see from Figure 9 that among all the cities in Finland, Helsinki has the strongest connection with Tampere, followed by Turku and Kuopio. Interestingly, Helsinki and Tampere have the same number of connected nodes (both cities are connected to all other cities) depicting that both cities are equally collaborative with other cities. An explanation for this could be given by the size of the cities since the capital city Helsinki is the largest city in Finland and Tampere the second largest in population size.

#### 3.6.3. Gene Set Enrichment Analysis

In this section, we are looking beyond single genes as, e.g., studied in Figure 7, and investigate genes collectively. Specifically, we are interested if genes studied globally are the same as the genes studied in Finland. That means we want to know if the group of highly studied genes outside of Finland corresponds to the genes that are also studied in Finland. In order to formalize this question, we perform an enrichment analysis.

In our enrichment analysis, we investigate the portion of genes which are studied in the whole world to see whether they are enriched or not in the three most prolific cities of Finland, see Table 1. In order to estimate the enrichment for a list of genes we need to assign two attributes to each gene. The first attribute, we call “HS” (highly studied), is having the two levels, “yes” and “no.” The second attribute, we call “City,” is having the two levels, “in” and “out.”

Specifically, our procedure works the following way. First, we are rank ordering all genes that have been studied worldwide by their number of appearances in the publications. Second, we group this list of genes into two subcategories by introducing a threshold γ. If the number of appearances in publications for a gene is above this threshold, we place this gene in category “HS-yes,” otherwise in category “HS-no.” That means we are distinguishing between genes that have been highly studied or not. Third, we give each gene a second attribute, “City.” If a gene has been studied in a specific “City” we give the gene the label “City-in,” otherwise “City-out.” That means we are distinguishing between genes that have been studied in a specific city or not. As a result, each gene has now two attributes based on which we base our enrichment analysis (see Methods section for details).

The Null Hypothesis we are studying for each city Y can be formulated by the following statement:

H_0_: The probability for a gene to be declared highly studied (“HS-yes”) and in city Y “City-In” is the same as the probability for a gene to be declared highly studied (“HS-yes”) and not in city Y “City-Out?”

**Figure 10:**
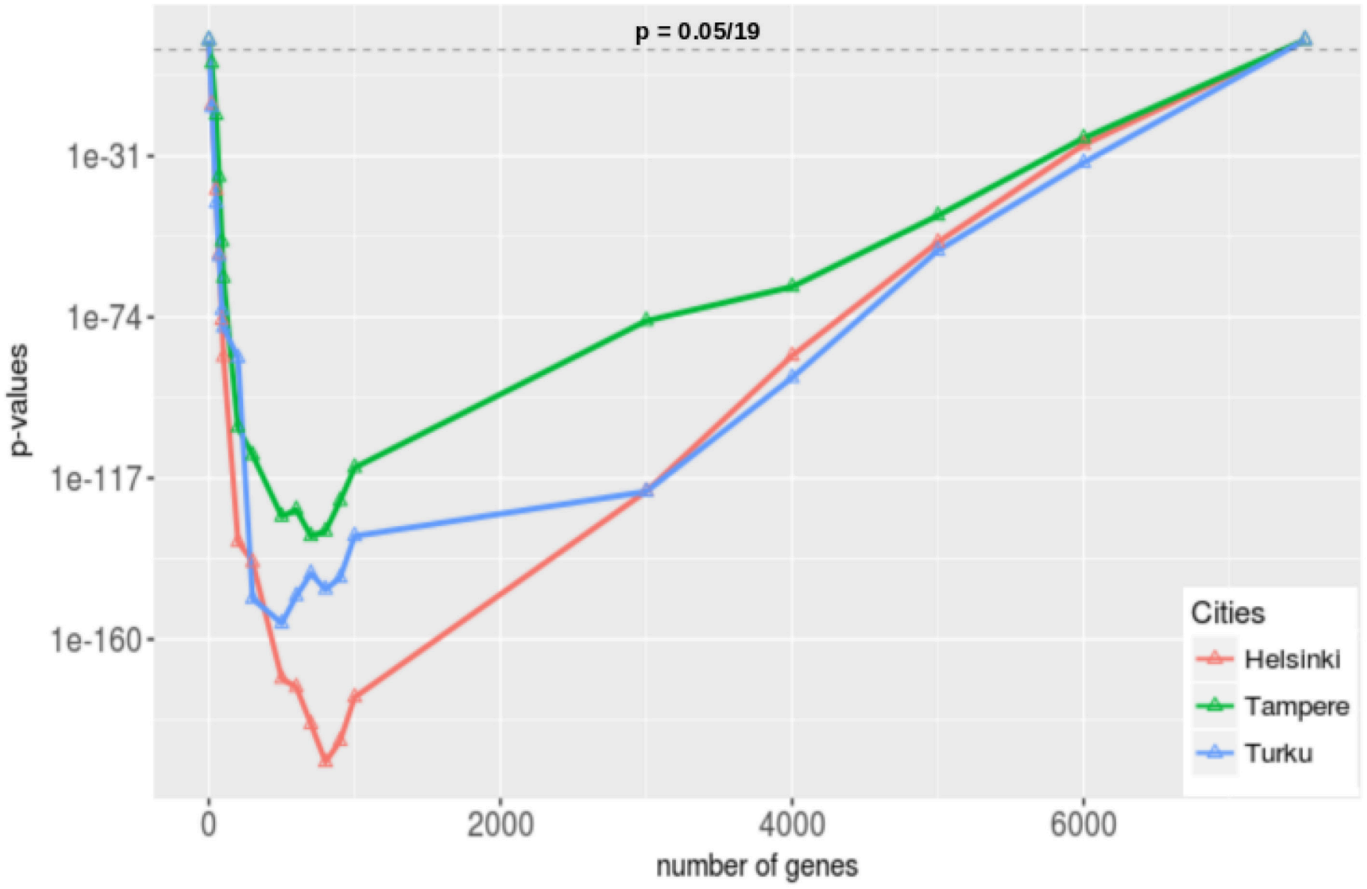
Gene set enrichment analysis for the three cities in Finland that publish most articles in prostate cancer research (see Table 1). We apply a Bonferroni correction because we are testing 19 hypothesis (one hypothesis for one threshold, γ) simultaneously.

The above procedure contains exactly one parameter, γ, for defining what we mean by highly studied genes. Because the correct value for this parameter is unknown, we use 19 different thresholds, 7,519, 6,000, 5,000, 4,000, 3,000, 1,000, 900, 800,700, 600, 500, 300, 200, 100, 90, 70, 50, 20, 0, and repeat our analysis for each of these parameters. Here 7519 corresponds to the total number of genes studied worldwide. In Figure 10, we show the results of our analysis for the three cities in Finland that publish most articles according to Table 1, Henslinki, Tampere, and Turku. The horizontal dashed line corresponds to the significance level of α = 0.05/19 we used to declare significance. We would like to note that we apply a Bonferroni multiple testing correction because we repeated the analysis 19 times. This provides conservative estimates.

From Figure 10, we see that in the two extreme cases, when no gene has been selected or when all the genes have been selected, nothing was enriched for all three cities as the corresponding p-values are 1. On the other hand, for all other gene sets that have been selected for different threshold values of γ, these gene sets are enriched for all three cities. From this we conclude that the researchers from Helsinki, Tampere, and Turku have very similar interests compared to the researchers from the rest of the world in the field of genetic prostate cancer research. Given the very low p-values (see the scale of the y-axis in Figure 10) ranging from 10^−31^ down to 10^−160^, these results are not sensitively dependent on the significance level and, hence, are very robust.

If one combines these findings with our results regarding the collaborations between Finland and other countries (see Figure 6) one wonders why the number of self-collaborations are so high given the fact that the genes studied in Finland are studied in other countries too? It seem that there is a lot of unused synergy that could be utilized in the future by establishing new international collaborations. Alternatively, one could also try to reduce the overlap in studied genes by specializing into a small number of unique genes to Finland. This would lead to unique features for Finland.

#### 3.6.4. Similarity of Research Among Cities

Finally, we study the similarity of prostate cancer research between the Finnish cities. For this reason, we are using hierarchical clustering with Ward's method and an Euclidean distance. As data we use the publication counts for the genes and the cities, i.e., we are using a matrix *M* where *M*_*ij*_ ∈ ℕ gives the number of publications for gene *i* and city *j*. In order to reduce noice from the data we focus on the top 19 genes most frequently studied worldwide.

**Figure 11:**
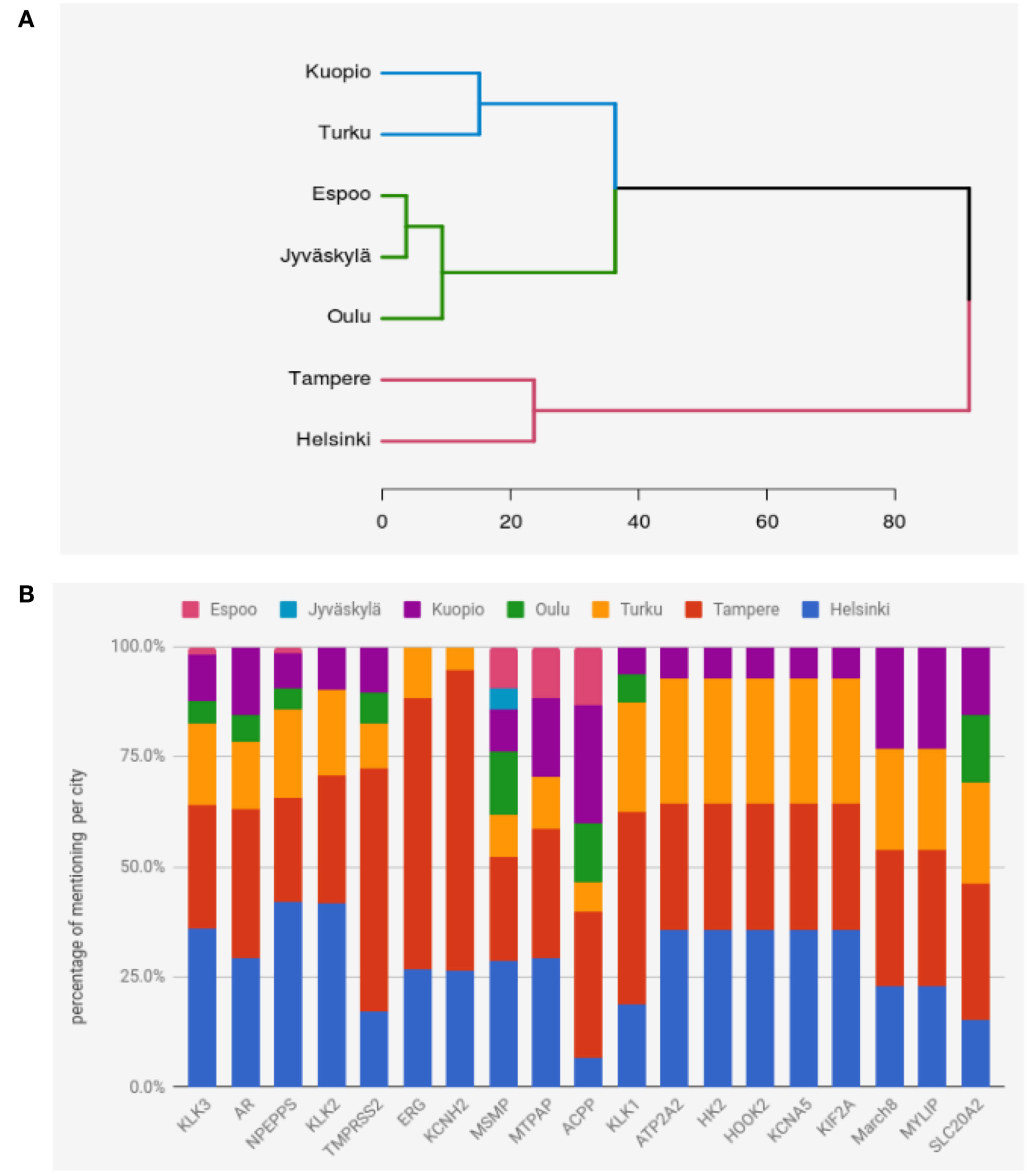
**(A)** Hierarchical clustering of cities in Finland showing their similarity of research interests in prostate cancer related genes. The results are based on the top 19 genes. **(B)** Proportions of mentioning of the top 19 genes in publications by the cities of Finland.

In Figure 11A, we show the hierarchical clustering for seven cities in Finland, see Table 1. Overall, the hierarchical clustering shows three clusters. One cluster with Tampere and Helsinki, the second with Kuopio and Turku and the third one with Espoo, Jyväskylä, and Oulu. Our interpretation of the clusters is that the cities within these have a common interest for similar genes. Furthermore, one observes that the two latter clusters are closer to each other and can be merged leaving only two major clusters. The similarity between Helsinki and Tampere in a cluster complements our findings about the collaborations between the cities, see Figure 9, where Helsinki and Tampere were connected with more links than other cities.

We were also interested to see whether certain cities had a greater interest in some specific genes compared to other cities. For this analysis we used the same top 19 genes as for the hierarchical cluster. For these top 19 genes, the number of publications for each gene ranges from 11 to 107.

In Figure 11B, we show the proportions of mentioning of the top 19 genes in publications by the cities of Finland. Except for ACPP and SLC20A2, Helsinki and Tampere contribute always more than 50% to all published articles for all genes. Interestingly, ERG and KCNH2 are exclusively studied by Helsinki, Tampere and Turku. The three genes KLK2, NPEPPS and KLK3 were mostly studied in Helsinki, while TMPRSS2, ERG and KCNH2 were mostly studied (around 60%) in Tampere. The second most studied gene in whole Finland is AR which was found in similar proportions in research papers of Helsinki and Tampere. We would like to note that Jyväskylä and Espoo have conducted the least number of studies on those top 19 genes.

## 4. Conclusion

In this paper, we provided a global overview of prostate cancer research over more than 30 years based on text mining publications in PubMed. We showed results for different levels including genes, countries, and cities in Finland and connections between those utilizing a computational network theory approach.

In terms of absolute numbers, the USA contribute by far the most number of publications to prostate cancer research in general and to genetics (see Figure 4). This is about six times more than all following countries, e.g., China or Germany. We further found that genetics research assumes a large portion of the total research in prostate cancer. Averaged over all countries 60% of all papers are about the genetics of prostate cancer. Interestingly, for China we observed 82% which is much higher than for all studied countries. This could indicate a different research strategy China is implementing by placing more focus on genetics research.

In order to see if and how the research in genetics changed over the years we studied the trends in the percentage of genetics research (see Figure 4B). Interestingly, we found that there are two periods behaving fundamentally different. For the first period we found a significant increase in the percentage of genetics research between 1987–1996 and 1997–2006 for most of the countries and all countries together. In contrast, for the second period between 1997–2006 and 2007–2018 we found a continuation of the previous levels and even a slight decline for some countries, although not significant. This indicates that the completion of the human genome project in 2003 did have a stimulating effect on genetics research in prostate cancer.

Regarding global collaborations in prostate cancer research, we found the USA to be the single most favorite collaboration partner for all countries, see Figure 5. Furthermore, China has the most homogeneous collaboration patterns, which means that this country has essentially only one collaboration partner, namely the USA. All other countries show heterogeneous patterns by favoring some countries over others. For Finland, we found similar results, see Figure 6.

Interestingly, the percentage of self-collaborations for all studied countries are above 75% (see Figures 5, 6) which means only one-quarter of their research has been done with international collaborations in the field of prostate cancer research in genetics. Japan has an even higher self-collaboration rate reaching 89%. Also this could point to a different research strategy, similar to China discussed above, by the health authorities in Japan.

From investigating the time evolution of studied genes, we found that many genes have been studied over the last 30 years, globally and in Finland, but there are only a few genes that have been studied much more frequently than all others. Specifically, these genes are KLK3, NPEPPS, and AR and, interestingly, they are the same globally and in Finland. Overall, the results shown in Figures 7A,B could be especially of interest for translational researchers interested in applications ranging from genetics to public health management and health policy because the provided information can directly inform patient-related decisions.

When studying the collaborations within Finland, we found Helsinki as the dominating hub followed by Tampere. Both, in terms of the number of collaborations as well as in the connectedness to other cities, see Figure 9. Another interesting result we found is that in Finland and in the rest of the world researchers have addressed a similar set of genes over the timeline of prostate cancer research from 1987 to 2018, see Figure 10. Importantly, this is independent of the exact determination of the threshold for defining the set of highly studied genes and, hence, is statistically robust (see Figure 10). From a biological point of view, we think the significant overlap in highly studied genes reflects the nature of prostate cancer as a complex disease (not as a Mendelian disease) (Botstein and Risch, 2003; Loscalzo et al., 2007; Altshuler et al., 2008). A complex disease can naturally not be understood by a single gene or a very small number of genes, but the interplay between many genes forming pathways and regulatory networks (Barabási, 2007; Emmert-Streib, 2007; Hopkins, 2008; Schadt, 2009; Emmert-Streib and Glazko, 2011). However, this leads naturally to genomics studies investigating the whole genome instead of individual genes. In this respect, our study detected this change in mindset in basic research from genetics to genomics despite the fact that individual genes are highlighted in separate publications. This is interesting because by considering many publications each focusing on a small number of genes one obtains results for many genes.

The last finding highlights the benefits of a meta-analysis as performed in our study because by collecting thousands of articles we could address questions that cannot be addressed by any single article individually (Kitchenham, 2004; Moher et al., 2009). We believe that the presented framework and findings could be helpful for researchers and cancer research laboratories all over the world including personalized medicine and public health management (Emmert-Streib and Dehmer, 2018).

## Author Contributions

FE-S conceived the study. MFA and AM performed the analysis. All authors wrote the paper and approved the final version.

### Conflict of Interest Statement

The authors declare that the research was conducted in the absence of any commercial or financial relationships that could be construed as a potential conflict of interest.
